# Evaluating the Experiences of New and Existing Teledermatology Patients During the COVID-19 Pandemic: Cross-sectional Survey Study

**DOI:** 10.2196/25999

**Published:** 2021-05-05

**Authors:** Judy Hamad, Amy Fox, Maria Suzanne Kammire, Alison Nancy Hollis, Saif Khairat

**Affiliations:** 1 Department of Dermatology School of Medicine University of North Carolina at Chapel Hill Chapel Hill, NC United States; 2 Carolina Health Informatics Program University of North Carolina at Chapel Hill Chapel Hill, NC United States; 3 Healthcare Systems School of Nursing University of North Carolina at Chapel Hill Chapel Hill, NC United States

**Keywords:** teledermatology, telehealth, patient satisfaction, patient-centered outcomes, COVID-19, dermatology, implementation, virtual health, digital health, cross-sectional

## Abstract

**Background:**

As teledermatology has been widely adopted during the COVID-19 pandemic, it is essential to examine patients’ experiences and satisfaction with teledermatology.

**Objective:**

We aimed to assess the teledermatology experiences of new and existing clinic patients in the context of the rapid shift toward teledermatology practices during the COVID-19 pandemic.

**Methods:**

We conducted a cross-sectional study of 184 teledermatology patients who were assessed during the COVID-19 pandemic at a major southeastern medical center from May 13 to June 5, 2020. The primary outcome was patient satisfaction levels among new and existing patients. The secondary outcome was patients’ willingness to use teledermatology in the future.

**Results:**

Of the 288 teledermatology patients who were assessed during the study period, 184 (63.9%) completed the survey. Patients reported high overall satisfaction with teledermatology, with 86.4% (159/184) of participants reporting positive overall satisfaction and experiences with teledermatology. New patients had significantly higher Likert scores for overall satisfaction with teledermatology than those of follow-up patients (new patients: mean 4.70; existing patients: mean 4.43; *P*=.03). Overall, patients’ satisfaction with teledermatology did not significantly differ based on age (*P*=.36), race and ethnicity (*P*=.46), education level (*P*=.11), residence (*P*=.74), or insurance status (*P*=.74). There were no significant differences in overall satisfaction between patients with and without prior telehealth experience (*P=*.53), between the video and telephone visit types (*P*=.17), and among platform types (*P=*.22). Prior telehealth experience was associated with higher odds of being willing to use telehealth in the future (odds ratio 2.39, 95% CI 1.31-4.35; *P*=.004).

**Conclusions:**

This cross-sectional survey study demonstrates that during the rapid expansion of teledermatology, new clinic patients had significantly higher scores for overall satisfaction with their teledermatology experience compared to those of established clinic patients (*P*=.03). Prior telehealth experience was associated with higher odds of being willing to use teledermatology in the future. Overall, teledermatology expansion was met with high levels of patient satisfaction during the COVID-19 pandemic.

## Introduction

With the rapid shift toward converting office-based dermatology clinics into web-based clinics during the COVID-19 pandemic [1,2], teledermatology has been increasingly used in clinical practice and has been a common subject of scientific literature in the past year [3]. Prior to the continuation of widespread teledermatology implementation, it is imperative that dermatologists examine patients’ experiences with teledermatology. The exchange of information through video, audio, and imagery has made it possible for dermatologists to visualize, diagnose, and communicate with patients throughout the pandemic. This rapid evolution has also resulted in the recognition of web-based services by most health insurance organizations [4,5] and the recording of telemedicine encounters in electronic health records. These changes will allow teledermatology to remain a prominent communication method in the future of the field.

Patients’ experiences likely differ based on patient-provider relationships and whether patients are new clinic patients or established clinic patients. Although some studies have reported high patient satisfaction after the use of teledermatology for new referrals or consults [6-9], to our knowledge there are no studies that examine new and existing patients’ satisfaction with teledermatology. Our objective was to assess new and existing patients’ satisfaction with teledermatology in the context of the COVID-19 pandemic–related rapid shift toward teledermatology practices.

## Methods

### Study Design

We conducted a cross-sectional study of teledermatology patients’ satisfaction during the COVID-19 pandemic at a major southeastern medical center. The rationale for this quality improvement initiative was to characterize patients’ experiences with teledermatology in order to improve our delivery of this mode of care. We reported this study per the Standards for Quality Improvement Report Excellence 2.0 guidelines [10]. This study was exempt from institutional review board approval due to its quality improvement objectives.

### Study Materials and Participants

Eligible participants were new and existing patients who attended teledermatology visits for acute and chronic conditions. Patients were invited to complete a postvisit survey, which was a voluntary survey that was adapted from a validated telehealth satisfaction study [11]. We recorded survey responses by using the web-based survey tool Qualtrics. We piloted the survey among 22 patients and made iterative survey changes based on patients’ and service providers’ feedback. We included responses from the piloted survey in the analysis, as there were minimal survey changes. We administered the survey from May 13 to June 5, 2020, to the patients of 8 dermatologists who delivered adult and pediatric teledermatology services. All patients who used adult and pediatric teledermatology services during the study period were eligible for inclusion in this study; however, the parents or guardians of pediatric patients completed the survey.

### Survey Questions

The survey instrument was adapted from the Telehealth Satisfaction Scale and modified by a telehealth domain expert so that the survey met the needs of this study. The survey consisted of 25 questions that addressed demographics, visit characteristics, and satisfaction measures (Multimedia Appendix 1). Patients reported whether they were new or existing patients of the clinic, whether they were telehealth-experienced patients or telehealth-naïve patients (no prior telehealth experience), and whether they were willing to use telehealth in the future (answered “yes” or “no”). Patients rated their satisfaction with 12 items on a Likert scale (1=poor; 2=fair; 3=good; 4=very good; 5=excellent). Satisfaction-related items included patients’ overall satisfaction with teledermatology, patient-related outcomes (personal comfort with teledermatology, the ease of using teledermatology, and respect for patients’ privacy), the voice and visual quality of the visit, time characteristics (the length of wait time and the length of time with the service provider), and service provider–related outcomes (treatment explanations, thoroughness, and the courtesy of the provider).

### Outcomes

The primary outcome was satisfaction levels among new and existing patients. The secondary outcome was patients’ willingness to use teledermatology in the future.

### Statistical Analysis

Satisfaction ratings of very good and excellent were considered positive ratings. Continuous measures were reported as means with SDs. Categorical variables were reported as numbers and percentages. Fisher exact tests were used for categorical variables, and *t* tests and one-way analysis of covariance tests were used to determine differences in the means of continuous variables. Univariable logistic regression was conducted to identify predictors of willingness to use teledermatology in the future. Statistical analyses were performed using Stata 16 (StataCorp LLC). A *P* value of <.05 was considered statistically significant.

## Results

### Study Population Characteristics

Of the 288 teledermatology patients assessed, 184 (63.9%) completed the survey (Table 1). The mean age of participants was 37.8 years, and 72.8% (134/184) of participants were females. Most teledermatology visits were conducted for existing patients (123/184; 66.8%) and telehealth-naïve patients (107/184; 58.2%). Most respondents were White (114/184; 62.0%) and had a Bachelor’s degree or other higher education degree (92/184; 50%). The majority of the respondents were privately insured (109/184; 59.2%), a large subset of patients had public insurance (69/184; 37.5%), and a minority of patients were uninsured (6/184; 3.3%).

**Table 1:** Participants’ characteristics (N=184).

Characteristic		Value
Age (years), mean (SD)		37.8 (18.3)
**Sex, n (%)**		
	Male	50 (27.2)
	Female	134 (72.8)
**Race, n (%)**		
	White	114 (62)
	Black/African American	44 (23.9)
	Hispanic/Latino	12 (6.5)
	Asian/Pacific Islander	10 (5.4)
	Other	4 (2.2)
**Education level, n (%)**		
	Less than high school	21 (11.4)
	High school or equivalent	32 (17.4)
	Some college	28 (15.2)
	Associate degree	11 (6)
	Bachelor's degree	46 (25)
	Graduate, doctorate, or professional degree	46 (25)
**Residence, n (%)**		
	Urban	47 (25.5)
	Suburban	87 (47.3)
	Rural	50 (27.2)
Unique patient zip codes reached, n		84
**Insurance status, n (%)**		
	Uninsured	6 (3.3)
	Private insurance	109 (59.2)
	Medicare, Medicaid, or Tricare	69 (37.5)
**Impairments, n (%)**		
	Visual	29 (15.8)
	Auditory	5 (2.7)
	Both	3 (1.6)
	None	147 (79.9)
**Patient type, n (%)**		
	New	61 (33.1)
	Existing	123 (66.8)
**Prior telehealth experience, n (%)**		
	Yes (telehealth-experienced patient)	77 (41.8)
	No (telehealth-naïve patient)	107 (58.2)
**Visit type, n (%)**		
	Video	171 (92.9)
	Telephone	13 (7.1)

### Patient-Reported Satisfaction

**Figure 1:**
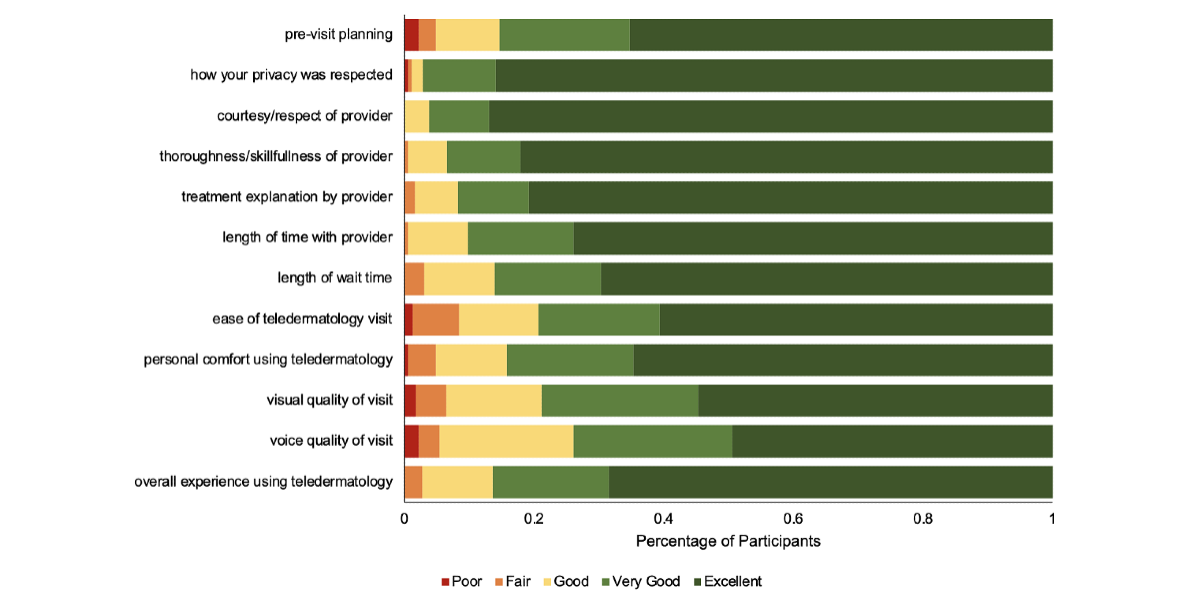
Patient-centered satisfaction outcomes following the completion of teledermatology visits.

**Figure 2:**
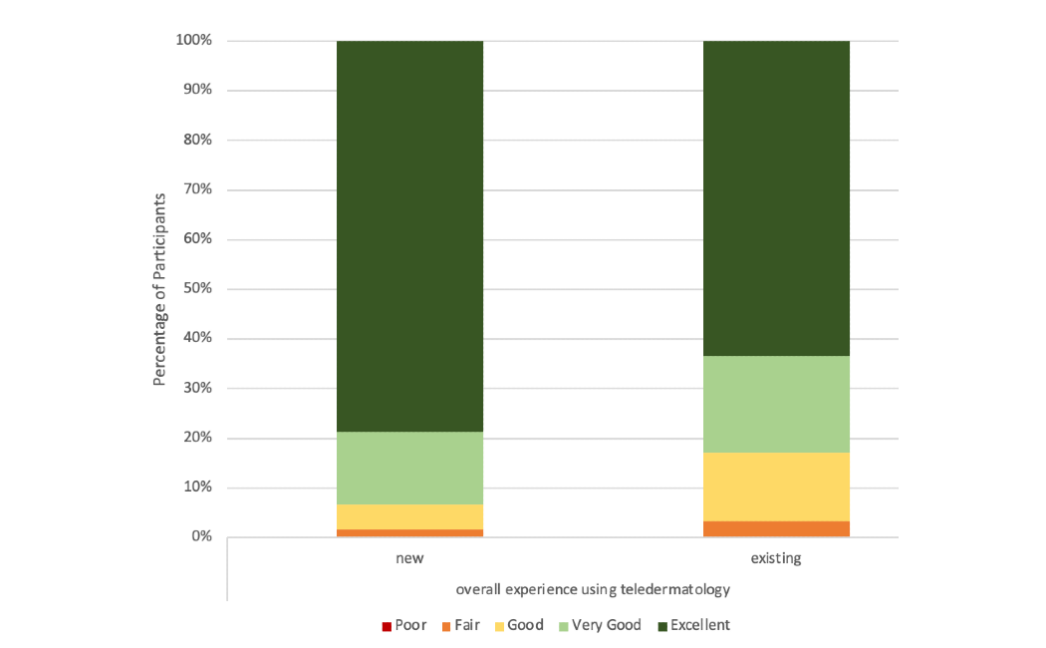
New and follow-up patients' overall satisfaction with teledermatology.

Patients reported high overall satisfaction with teledermatology (Figure 1), with 86.4% (159/184) of participants reporting a positive teledermatology experience. New patients had significantly higher Likert scores for overall satisfaction with teledermatology than those of follow-up patients (new patients: mean 4.70; follow-up patients: mean 4.43; *P*=.03). Patients’ satisfaction with teledermatology did not significantly differ based on age (*P*=.36), race and ethnicity (*P*=.46), education level (*P*=.11), residence (*P*=.74), or insurance status (*P*=.74). There were no significant differences in overall satisfaction between patients with and without prior telehealth experience (*P*=.53) and between the video and telephone visit types (*P*=.17). In terms of all of the satisfaction measures, new patients reported higher satisfaction scores compared to those reported by existing patients; however, these differences were not statistically significant (Figure 2).

**Figure 3:**
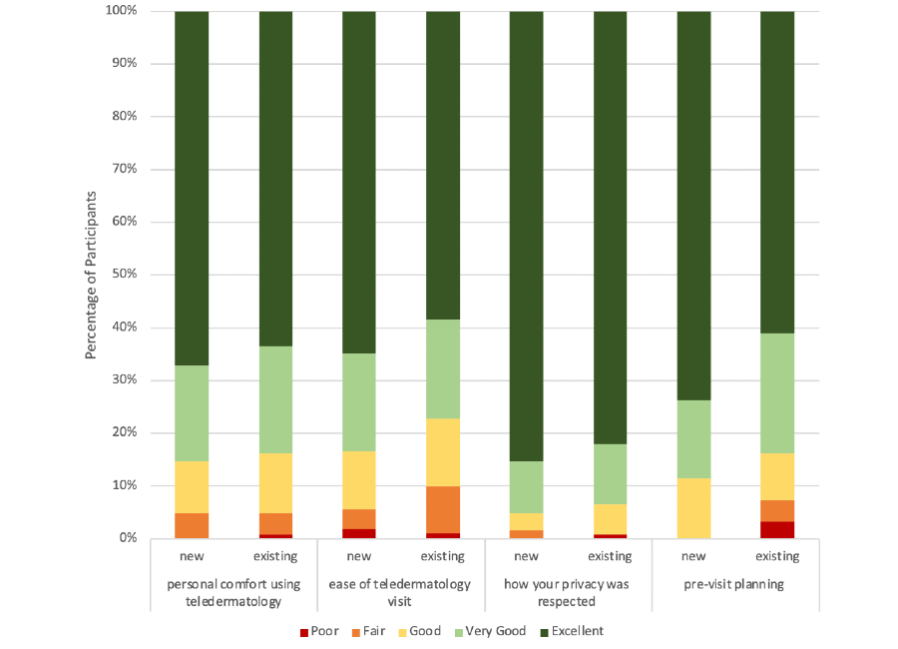
New and follow-up patients' satisfaction with the following patient-related outcomes: comfort, ease, privacy, and previsit planning experiences.

Patients’ personal comfort with using telehealth and the ease of using telehealth were similar between new and follow-up patients (Figure 3). Participants reported high satisfaction with the privacy of telehealth visits, with 85.2% (52/61) of new patients and 82% (100/122) of follow-up patients rating their satisfaction as “excellent” (Figure 3). Patients’ satisfaction with previsit planning was different between the two groups (*P*=.15); follow-up patients reported lower levels of satisfaction (excellent: 75/123, 61%; very good: 28/123, 22.8%; good: 11/123, 8.9%; fair: 5/123, 4.1%; poor: 4/123, 3.3%), while new patients reported slightly higher levels of satisfaction with the teledermatology process (excellent: 45/61, 73.7%; very good: 9/61, 14.8%; good: 7/61, 11.5%; fair and poor: 0/61, 0%; Figure 3). Participants’ overall satisfaction with the voice quality of visits was low, and follow-up patients’ satisfaction with voice quality was lower than new patients’ satisfaction. Patients’ satisfaction with visual quality was slightly higher than their satisfaction with voice quality and similar between follow-up patients and new patients. The length of wait time, length of time with the service provider, and provider-related satisfaction were highly rated among participants.

### Willingness to Use Teledermatology in the Future

Our univariable logistic regression showed that prior telehealth experience was associated with higher odds of being willing to use teledermatology in the future (odds ratio [OR] 2.39, 95% CI 1.31-4.35; *P*=.004). Age, sex, race, education, residence, and insurance status were not associated with significant odds of preferring teledermatology (Table 2). Compared to new patients, existing patients had nonsignificantly higher odds of using of telehealth in the future (OR 1.46, 95% CI 0.79-2.72; *P*=.23).

**Table 2:** Univariate logistic regression results for predicting patient’s willingness to use teledermatology in the future.

Predictor			Preferred telehealth, odds ratio (95% CI)		*P* value
**Age (years)**					
	<18	Referent		N/A^a^	
	18-34	1.51 (0.54-4.24)		.44	
	35-64	1.14 (0.40-3.25)		.80	
	≥65	0.42 (0.11-1.55)		.11	
**Sex**					
	Female	Referent		N/A	
	Male	1.16 (0.61-2.23)		.65	
**Race**					
	White	Referent		N/A	
	Black/African American	1.19 (0.59-2.39)		.62	
	Other	1.19 (0.51-2.80)		.69	
**Education level**					
	Less than high school	Referent		N/A	
	High school or equivalent	0.62 (0.21-1.89)		.40	
	Some college	0.43 (0.13-1.38)		.16	
	Associate degree	0.20 (0.03-1.17)		.08	
	Bachelor's degree	1.41 (0.50-4.01)		.51	
	Graduate, doctorate, or professional degree	0.99 (0.35-2.79)		.99	
**Residence**					
	Rural	Referent		N/A	
	Suburban	1.23 (0.61-2.48)		.56	
	Urban	1.57 (0.70-3.50)		.70	
**Insurance status**					
	Medicare, Medicaid, or Tricare	Referent		N/A	
	Private insurance	1.2 (0.66-2.20)		.55	
	Uninsured	0.61 (0.11-3.57)		.59	
**Patient type**					
	New	Referent		N/A	
	Follow-up	1.46 (0.79-2.72)		.23	
**Prior telehealth experience**					
	No	Referent		N/A	
	Yes	2.39 (1.31-4.35)		.004	

^a^N/A: not applicable.

## Discussion

### Principal Findings

We conducted an evaluation of teledermatology implementation as a response to the COVID-19 crisis. Although there is a limited number of prior teledermatology studies that evaluate patients’ satisfaction, this study found that patients’ satisfaction was high across numerous key measures. We found that 97.3% (179/184) of patients reported a positive overall experience with teledermatology (ratings of good, very good, or excellent). These findings are consistent with those of prior teledermatology studies in related literature [7-9]. Additionally, we found that patients’ overall satisfaction with telehealth did not vary significantly based on patients’ demographic characteristics, locations of residence, education, or insurance status.

Our results demonstrated that new patients had significantly higher overall scores for satisfaction with teledermatology than those of existing patients (*P*=.03). Furthermore, new patients reported higher satisfaction for all satisfaction metrics. This may be due to the fact that new patients did not have prior in-person experiences with the dermatologist that they were seeing; therefore, they could not compare different teledermatology experiences. Furthermore, existing patients may have been more inclined to compare their teledermatology visit to those they experienced in person. This high satisfaction among new patients could also be related to the fact that we reached out to geographically diverse patients across 84 zip codes who were enthusiastic about having increased access to dermatology services.

Interestingly, over half of our participants (107/184, 58.2%) never used telehealth services prior to their teledermatology visit during the COVID-19 pandemic. In an Italian study of teledermatology patients of an acne center that was conducted during the COVID-19 pandemic, patients reported favorable experiences, and 92% of patients appreciated their visits [12]. This was similar to our study. However, the Italian study’s patient population entirely consisted of existing patients. In contrast, we observed various conditions among the new and existing patients, especially among telehealth-naïve patients. We found that prior telehealth experience was associated with a willingness to use teledermatology in the future. The increasing awareness of telehealth benefits, such as time and cost savings, among patients with no telehealth experience may help mitigate people’s resistance to future telehealth use [13]. Additionally, because people compare prior in-person visits to teledermatology visits, our patients may have considered in-person visits to be more thorough than video visits. The deployment of teledermatology during our study period likely reflected the broader use of telehealth for conditions other than those that would be present after the COVID-19 pandemic and the need for disease-specific scheduling algorithms, which can ensure that the telehealth modality suits the target condition. However, the overall satisfaction of both new and follow-up patients was extremely positive, and the expansion of teledermatology services seemed to be well received by patients.

The impacts of the national health crisis have undoubtedly influenced patients’ perception of care and have likely influenced patients’ willingness to engage in teledermatology in a way that is unprecedented in prior studies. We suspect that at least a portion of the highly positive responses to teledermatology visits from our surveyed patients was due to teledermatology providing patients with the ability to avoid high-risk settings and continue to practice social distancing by staying at their homes [14]. In addition, many patients travel long distances to be seen by specialists at the clinic and are pleased to save time, money, and energy by not having to physically appear at clinics. It is for these reasons that the amplification of the role of telehealth has been regarded as a silver lining or “bright spot” of the pandemic [15-18]. Although the Centers for Medicare & Medicaid Services have provided payment parity for telehealth visits and service providers can bill patients for telehealth visits at the same rates as in-person visit rates, patients have likely saved money by taking less time off of work and not having to consider gas costs for trips to clinics.

### Limitations

The findings of our research are limited by the nature of our study. First, this study reported findings from a single cross-sectional sample of patients who were treated by 8 participating dermatologists. Although patients were recruited consecutively at the end of telehealth visits, several patients did not stay on the phone or video call to immediately complete the survey. It is possible that patients who did not complete the survey had characteristics that considerably differed from the characteristics of those who did complete the survey, given that we had a nonresponse rate of 36%. Participants in this study were more likely to be White; educated; insured; and, on average, younger than the general population. Although we did not find statistical differences in satisfaction based on the many demographic characteristics we analyzed, it is possible that a larger sample size would have resulted in the observation of important differences in satisfaction. One study reported a potential disparity—the decreased amount of video visit use among older adults [19]. This highlighted the following key questions: (1) which populations do or do not have access to telehealth, and (2) how does this impact disparities in care? Second, this study took place at a single dermatology department at an academic institution. Thus, we cannot generalize our results to the larger dermatology patient population. Third, we did not collect information regarding diagnosis; it is plausible that patients’ satisfaction with telehealth may differ based on dermatologic conditions that require more or less complicated management. Finally, we observed a ceiling effect in our data. Since satisfaction scores were rated on a Likert scale of 1-5, the ratings tended to be grouped at the higher end of the scale. This ceiling effect likely resulted in less variability among the data and limited our ability to test associations or build multivariable regression models.

### Conclusion

We report that the rapid expansion of teledermatology resulted in new patients reporting higher satisfaction with their teledermatology experiences compared to the satisfaction of existing patients of the clinic. Prior telehealth experience was associated with higher odds of being willing to use teledermatology in the future. The rapid adoption of teledermatology during the study period was met with high overall levels of patient satisfaction during the COVID-19 pandemic. The deployment of teledermatology during our study period likely reflected the broader use of telehealth for conditions other than those that would be present after the COVID-19 pandemic and highlighted the need for disease-specific scheduling algorithms, which can ensure that the telehealth modality suits the target condition.

## Appendix

Multimedia Appendix 1 Teledermatology patient satisfaction survey tool.

## Abbreviations

OR: odds ratio
