# Supplementary material for: Evaluating the Experiences of New and Existing Teledermatology Patients During the COVID-19 Pandemic: Cross-sectional Survey Study
Source: JMIR Dermatol. 2021 May 5;4(1):e25999. doi: 10.2196/25999 (PMC8104278; doi:10.2196/25999)
Supplement: Multimedia Appendix 1 [file derma_v4i1e25999_app1.pdf]

1. Are you the patient or parent/guardian/caregiver answering on behalf of the patient?
2. Age
3. Race/ethnicity
    a. White/Caucasian, Black/African American/African/Caribbean, Asian/Pacific Islander, Native America, Hispanic/Latino, or other
4. Gender identity
    a. Male, female, gender non-binary/non-conforming, other
5. Highest level of education completed
    a. Less than high school diploma, high school diploma or equivalent (e.g. GED), some college/no degree, associate degree, bachelor degree, or graduate/professional/doctorate
6. Residence
    a. Urban, suburban, or rural
7. Zip code
8. Hearing or visual impairments
    a. Hearing, visual, both, or none
9. Health insurance
    a. Medicare/Medicaid/Tricare, private insurance, or no health insurance
10. New or existing dermatology patient of this clinic
11. Prior experience with telehealth
12. How satisfied were you with:
    a. Your overall experience with teledermatology
    b. Voice quality of visit
    c. Visual quality of visit
    d. Personal comfort using teledermatology
    e. Ease of the teledermatology visit
    f. Length of wait time
    g. Length of time with the provider
    h. Explanation of treatment by provider
    i. Thoroughness/skillfulness of provider
    j. Courtesy/respect of provider
    k. How well your privacy was respected
    l. How well-supported you felt in advance of your visit by the pre-visit planning with nursing/office staff
13. For your next visit, would you prefer:
    a. in-clinic visit, teledermatology visit, or no preference
14. Describe favorable reasons for choosing teledermatology (open-ended)
15. Describe any challenges or barriers to using teledermatology (open-ended)
